# The Influence of Anesthesiologist Gender and Experience on Risk Understanding and Anxiety Changes After Online Preoperative Patient Education: A Sub-Analysis of the iPREDICT Randomized Controlled Trial

**DOI:** 10.3390/jcm14217643

**Published:** 2025-10-28

**Authors:** Alma Puskarevic, Heidi Ehrentraut, Andrea Kunsorg, Izdar Abulizi, Andreas Mayr, Milan Jung, Maximilian Schillings, Caroline Temme, Annika Pütz, Mark Coburn, Maria Wittmann

**Affiliations:** 1 Department of Anesthesiology and Intensive Care Medicine, University Hospital Bonn, 53127 Bonn, Germany; heidi.ehrentraut@ukbonn.de (H.E.); andrea.kunsorg@ukbonn.de (A.K.); milan.jung@posteo.de (M.J.); s4maschi@uni-bonn.de (M.S.); s4catemm@uni-bonn.de (C.T.); annika.puetz@ukbonn.de (A.P.); mark.coburn@ukbonn.de (M.C.); maria.wittmann@ukbonn.de (M.W.); 2 Department of Medical Biometry and Statistics, University of Marburg, 35032 Marburg, Germany; abulizi@uni-marburg.de (I.A.); andreas.mayr@uni-marburg.de (A.M.)

**Keywords:** gender, clinical experience, preoperative care, anesthesia risks, online education

## Abstract

**Background/Objectives:** Digital health technologies are increasingly integrated into perioperative care to standardize information delivery and improve patient empowerment. However, the overall effectiveness of preoperative education depends not only on digital tools but also on interpersonal factors, such as physician gender and clinical experience, which may shape patients’ perceptions and responses to digitally delivered content. **Methods:** Patients scheduled for elective surgery were included in the iPREDICT randomized trial prior to their preoperative anesthesia assessment. After preoperative anesthetic assessment, the anesthesiologist documented the communication quality and the risks explained. Patients completed a questionnaire to assess their knowledge of anesthesia-related risks and whether the consultation alleviated their fears. **Results:** A total of 275 included patients were consulted by 94 anesthesiologists, 65% of whom were female. Risk recall was mainly determined by patient-related factors, with online education significantly improving recall over time (β = 1.24, *p* = 0.034). Anesthesiologists with 1–4 years of clinical experience explained more risks than those with <1 year of professional experience (β = 2.30, *p* = 0.024). A reduction in post-consultation anxiety was noted when the anesthetist was female (β = 0.21, *p* = 0.022). Communication was overall rated as good, with higher ratings when anesthetists had more than 10 years of experience (β = 0.09, *p* = 0.049). **Conclusions:** Although we have shown with the iPREDICT study (registered in the German CTS; DRKS00032514; on 21 August 2023) that online education improves patients’ recall of anesthesia-related risks, the current sub-analysis emphasizes that interpersonal interactions remain essential for alleviating fears and improving the quality of communication. Together, these findings underscore the complementary roles of digital education and face-to-face consultations in optimizing preoperative preparation.

## 1. Introduction

With the increasing number of elective surgeries, optimizing preoperative preparation has become a priority [1,2]. Consequently, preoperative patient management is evolving rapidly and has become an essential component of patient-centered perioperative care [3,4]. This optimization is particularly relevant in the field of anesthesia, where the provision of clear and comprehensive information is crucial to ensure trust and to help the patient reach an informed decision [5]. During the preoperative anesthetic assessment before surgery, the patient receives general information about anesthesia, their concerns are addressed, and specific information about their health status is obtained [3]. Quality communication in the preanesthetic clinic has been shown to reduce patient anxiety, improve adherence to fasting and medication instructions, and enhance overall satisfaction with perioperative care [6,7]. Effective communication about treatment options and care plans is an essential part of shared decision-making, particularly when these decisions affect a patient’s health status [7,8].

In this context, digital education tools are being increasingly integrated into healthcare to standardize and clarify information delivery and promote patient empowerment [9,10]. Understanding how clinician-related factors interact with digital education is essential to ensuring equitable and effective patient preparation across diverse healthcare settings. However, the overall effectiveness of preoperative education depends not only on digital tools but also on the interpersonal interaction with the physician, especially during face-to-face visits, where factors such as physician gender and clinical experience may influence how patients respond to previously conveyed digital content. Previous studies have shown that gender differences among healthcare providers can influence satisfaction, visit duration, and outcomes of surgical patients [11,12,13]. Patients’ expectations of physicians may also vary based on the physician’s gender [14]. Similarly, research in clinical and training settings indicates that physician experience can enhance communication quality over time [15,16]. Unlike in other specialties, anesthesiologists often meet patients only once before surgery, making the quality of this single interaction particularly important. Despite these insights, few studies have specifically investigated how an anesthesiologist’s gender and clinical experience influence patients’ preoperative preparation [17].

In our initial prospective, randomized, placebo-controlled iPREDICT study, we demonstrated that online education prior to an in-person preoperative visit significantly improves patients’ knowledge of anesthesia-related risks and promotes patient empowerment [18]. Building on these findings, the present sub-analysis aimed to explore the role of interpersonal factors, specifically the gender of the anesthesiologist and level of clinical experience, in shaping patients’ preoperative preparation within the context of digital education. More specifically, this study examined whether individual physician characteristics influence patients’ understanding and recall of anesthesia-related risks, and whether anesthesiologist gender affects the reduction in patient anxiety after the face-to-face preoperative consultation. Furthermore, this sub-study aimed to clarify how these physician characteristics interact with digital education tools and influence communication quality in perioperative care.

## 2. Materials and Methods

**Figure 1:**
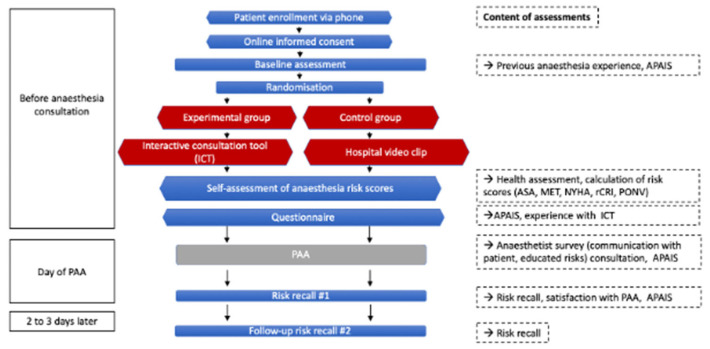
Flow diagram of the iPREDICT study.

The present sub-analysis investigated 275 adult patients who were scheduled for elective surgery under general anesthesia or combined regional and general anesthesia. They were recruited within the iPREDICT Trial from September 2023 to January 2025. The iPREDICT study was a prospective, randomized, placebo-controlled clinical study conducted at the University Hospital Bonn, Germany [18]. The study is registered in the German Clinical Trials Register (https://drks.de/search/en/trial/DRKS00032514; accessed on 21 August 2023) (Figure 1).

Participants were enrolled in the study prior to their preoperative anesthetic assessment at the clinic and randomly assigned to an online anesthesia risk education in an experimental ICT group (Interactive consultation tool group) or to a control group that watched a video without any anesthetic risk content. Details of the trial design and procedures have been reported previously; only methods relevant to the present sub-analysis are summarized here [18].

In addition to the baseline demographic and clinical data, data on the patient’s anesthesia-related anxiety levels and information needs in the preoperative phase were collected using the six-point Amsterdam Preoperative Anxiety and Information Scale (APAIS) [19]. On the day of the preoperative consultation, anesthesiologists completed a structured questionnaire documenting the quality of communication, the specific risks explained, and whether the patient declined a detailed explanation. The anesthesiologists who conducted the consultations were randomly assigned to patients according to the clinic’s appointment schedule. They were unaware of the patients’ group assignment (ICT group vs. control) and completed a standardized questionnaire immediately after each consultation. Patients completed a separate questionnaire evaluating their knowledge of potential risks and whether the consultation had alleviated their fears and concerns. Two days later, patients were surveyed again to assess retention of anesthesia-related risk information.

### 2.1. Outcome Parameters

The primary outcome of this sub-analysis was patients’ understanding of the risks associated with anesthesia after personal consultation, as well as the influence of gender and the anesthetist’s clinical experience on this parameter. In this context, risk understanding was defined as the patient’s overall comprehension of anesthesia-related risks following both digital and in-person education; risk recall referred to the number of individual risks the patient could correctly remember at each assessment time point; and risk explanation reflected the number of risks communicated by the anesthesiologist during the consultation.

Secondary outcomes included changes in patients’ preoperative anxiety levels, concern about anesthesia, and information needs measured with the APAIS score at three time points: at the baseline visit, after online education, and after the personal consultation. In addition, patients’ perceptions of consultation-related relief of fears and concerns were assessed. Further exploratory outcomes were as follows: the duration of the clinical consultation, the frequency with which patients asked questions, the effectiveness of communication between the patient and the anesthetist, and whether the patient refused the detailed educational explanation.

### 2.2. Statistical Analysis

Descriptive statistics for key measures are stratified by the anesthesiologist’s gender, as well as the patient’s gender. Continuous variables are presented as medians with interquartile ranges (Q1, Q3), and categorical variables as numbers with percentages. To examine differences in patient-reported anxiety levels, information needs, understanding of anesthesia-related risks, and perceived communication effectiveness, we applied linear mixed-effects models (LMMs). Fixed effects included patient age, patient gender, anesthesiologist gender and clinical experience, study group, with random intercepts for anesthesiologist. For understanding of anesthesia-related risks, random intercepts are additionally included for patients to account for repeated measurements at up to two time points, immediately after the consultation and during the online follow-up two days later. A significance level of 0.05 was used for all statistical tests. Due to the exploratory nature of the study, no adjustment for multiple testing was performed. Analyses were performed using R Version 4.4.2 (R Foundation for Statistical Computing, Vienna, Austria).

## 3. Results

### 3.1. Study Cohort and Characteristics

The present analyses included 275 patients enrolled in the iPREDICT trial [18]. All patients completed the assigned online preoperative education module at home prior to their preoperative anesthetic consultation at the clinic. The median age of the study population was 57 years [Q1, Q3: 43.0, 67.0]. Female patients were younger on average (48 years) compared to male patients (60 years). The distribution of surgical risk categories and ASA (American Society of Anesthesiologists) physical status scores is presented in Table 1. Most patients were classified as having low (51%) or intermediate surgical risk (44%), with only a small proportion undergoing high-risk or cardiac surgery procedures (5%). Regarding physical status classification, the majority of patients were categorized as ASA II (65%), followed by ASA I (15%) and ASA III (19%), while only two patients (1%) were classified as ASA IV. The median ASA score was 2.0 [Q1, Q3: 2.0, 2.0], indicating that most patients had mild systemic disease.

A total of 94 anesthesiologists consulted 275 patients (Table 2). Most consultations were performed by anesthetists with 1–4 years of professional experience (40%), followed by those with <1 year (30%), >5 years (18%), and >10 years (12%). Female anesthetists conducted most consultations (65%), especially in the groups with less clinical experience, while in the group with more than ten years of experience, consultations were more frequently conducted by male anesthetists (61%).

### 3.2. Primary Analysis

On the day of the preoperative consultation, patients were informed about a median of 9 risks [Q1, Q3: 6.0–15.0] out of 15 possible risks (Table S1). There was no significant difference in the number of risks explained based on the gender of the patient or the gender of the anesthesiologist, with male and female patients receiving comparable information. Anesthesiologists with 1–4 years of experience explained significantly more risks compared to those with <1 year of experience (β = 2.30, 95% CI: 0.31–4.29, *p* = 0.024) (Table 3). Anesthetists with moderate experience (>5 years, β = 1.49, *p* = 0.208) reported approximately 1.5 more risks than those with <1 year of experience. Those with the most experience (>10 years, β = 2.36, *p* = 0.105) reported approximately 2.4 more risks than those with <1 year of experience, similar to the group with 1–4 years of experience, but not statistically significant. Participation in the ICT group (Interactive consultation tool group) did not influence how many risks were explained by anesthesiologists (β = 0.11, *p* = 0.853).

In contrast, variability in risk recall was almost entirely attributable to patient factors rather than anesthesiologist factors. Immediately after the consultation, patients in both groups recalled a median of 12 risks [Q1, Q3: 8.00, 15.0]. In an online follow-up questionnaire two days later, patients recalled a median of 13 risks [Q1, Q3: 10.0, 15.0] (Table S2).

This indicates that patient characteristics and the online education intervention were more important for memory retention than the person delivering the information. The linear mixed-effects model confirmed a significant interaction between time and group (β = 1.24, 95% CI 0.09–2.38, *p* = 0.034), demonstrating that online education significantly improved risk recall over time. At follow-up, patients in the ICT group recalled on average 2.11 risks more than those in the control group. Furthermore, increasing patient age was associated with lower recall (β = −0.04 per year, 95% CI −0.07 to −0.01, *p* = 0.005) (Table 4). Older patients had slightly more difficulty retaining detailed risk information. Neither the gender of the patient nor the gender of the anesthesiologist significantly influenced risk recall.

### 3.3. Secondary Analysis

We assessed preoperative anesthesia-related anxiety and the need for information regarding anesthesia at three different time points. The overall median APAIS (Preoperative Anxiety and Information Scale) score on consultation day was 2.00 [Q1, Q3: 1.67, 2.33], indicating generally low levels of preoperative anxiety in the study cohort. Male patients had a slightly lower median anxiety score of 2.00 [Q1, Q3: 1.67, 2.33] compared to female patients with 2.33 [Q1, Q3: 1.67, 2.67] (Table S3). A linear mixed effects model revealed that patient age correlated negatively with preoperative anxiety (β = −0.0066/y, 95% CI: −0.01 to −0.00, *p* = 0.036) (Table S4). Older patients were slightly less anxious than younger patients, although the effect was small. Female patients tended to report somewhat higher anxiety scores than male patients (β = 0.20, 95% CI: 0.01–0.41, *p* = 0.061). Whether patients received online preoperative information (ICT group) or standard information had no significant effect on anxiety levels (β = 0.04, *p* = 0.689). When considering the influence of the anesthetist, neither the gender nor the experience of the anesthetist had a significant influence on the patients’ anxiety levels.

In a further analysis, patients reported a reduction in anxiety after the consultation, with a median score of 4.00 [Q1, Q3: 3.00–4.00] on the four-point Likert scale, where higher values indicate greater relief of fears (Table S5). The gender of the anesthetist was the only significant predictor, with patients reporting slightly greater anxiety relief when the consultation was conducted by a female anesthetist (β = 0.21, 95% CI 0.03–0.38, *p* = 0.022) (Table 5). Furthermore, neither anesthesiologist experience, digital preparation, nor patient sex had an effect.

Other secondary outcomes measured after the consultation showed no differences between genders or groups. The only effect was observed in communication quality, which was rated higher by patients when the anesthetist had more than 10 years of experience (β = 0.09, 95% CI: 0.00–0.17, *p* = 0.049) (Table 6). Overall, patients asked a moderate number of questions and generally felt well informed, which was reflected in their low need for additional information. It should also be mentioned that a total of 39% of patients refused a detailed educational session, with similar proportions among men (39%) and women (38%) (Table S5).

## 4. Discussion

In this sub-analysis of the iPREDICT trial [18], we investigated the influence of anesthesiologist gender and clinical experience on preoperative patient preparation in the context of digital education. We demonstrated with the iPREDICT trial that online preoperative education prior to an in-person visit significantly improved patients’ knowledge of anesthesia-related risks over time. This sub-analysis found that anesthesiologist characteristics primarily affected aspects of communication and reassurance. Female anesthesiologists were more likely to alleviate patients’ fears, and anesthesiologists with 1–4 years of experience explained more risks compared to those with <1 year of experience. In contrast, communication quality was rated highest when anesthesiologists had more than 10 years of experience. Overall, preoperative anxiety was low, and recall of risks was largely determined by patient characteristics, particularly age, rather than anesthesiologist factors.

Given the exploratory nature of this analysis and the number of models tested, the results should be interpreted with caution. The wide confidence intervals for certain predictors suggest limited significance in some subgroups. The observed gender differences likely reflect complex communication and interpersonal factors rather than gender alone. However, our findings align with previous research suggesting that physician gender can influence patient satisfaction, trust, and communication quality [20,21]. Previous studies have shown that female physicians show more empathy and provide more psychosocial support, asking more questions and providing more information compared to their male colleagues [22]. This may explain why patients reported greater relief from fear when the consultation was led by a female anesthesiologist. Also, this finding should be interpreted with caution, given the gender imbalance in our sample. Most consultations were conducted by female anesthesiologists, which may have influenced the overall direction of the observed effects.

Similarly, the observation that anesthesiologists in their early years of training explained more risks is consistent with earlier work showing that younger clinicians may adhere more strictly to protocols and provide more comprehensive risk disclosure, while experienced practitioners rely more on judgment [23]. The group of anesthesiologists with 1–4 years of experience was the most thorough, probably because they have gained confidence, but still adhere strictly to protocol. Conversely, the higher communication ratings for anesthetists with longer professional experience probably reflect interpersonal skills acquired over time, even if detailed explanations of risks were less common. This pattern may reflect a trade-off between thoroughness and patient understanding: while young anesthesiologists provide detailed, protocol-oriented explanations, too much information can overwhelm patients [24]. In contrast, experienced physicians often focus on clarity and relevance, which can improve patient understanding and reassurance [15]. This clarity not only supports informed consent, but can also reduce perioperative stress and improve patient satisfaction—outcomes that are becoming increasingly important in modern anesthetic practice. The integration of an online education tool in the perioperative clinical practice proved significant advantages for patient empowerment and decision-making [18]. The variability in risk recall was almost entirely due to patient factors, not the anesthesiologist, indicating the ICT (Interactive consultation tool) was more important for retention than who delivered the information. Importantly, this effect was independent of anesthesiologist gender or experience, suggesting that digital tools can standardize the delivery of essential information. At the same time, our data highlight that interpersonal interactions remain crucial for reducing preoperative fears and shaping the patient’s perception of communication quality. Together, these findings suggest that digital tools should complement, rather than replace, face-to-face consultations in preoperative education. Integrating such tools into routine practice can enhance patient understanding, while interpersonal communication remains essential for reducing anxiety and promoting trust. Patients who obtain basic knowledge in advance through online education are also better prepared to engage in meaningful dialog with the anesthesiologist, ask specific questions, and participate more actively in shared decision-making, which also reflects greater patient empowerment.

Targeted communication training, particularly for less experienced anesthesiologists, could further improve patient interactions [25]. Future research should examine how combining digital preparation with structured communication approaches influences patient outcomes across diverse clinical and cultural settings. From a practical perspective, these findings underscore the importance of integrating digital education tools into structured communication training for anesthesiologists, particularly those early in their careers. Such training could help balance thoroughness and clarity, improve patient-centered communication, and ensure that digital and interpersonal approaches to preoperative care effectively complement each other. Integrating evidence-based digital tools into structured communication training could represent the next step toward a standardized yet individualized model of preoperative patient education.

### Limitations

This study has several limitations. First, its generalizability may be limited, as it was conducted in a single hospital in Germany with a moderate sample size derived from the iPREDICT trial. Furthermore, cultural norms and expectations regarding gender roles in physician–patient interactions may have influenced the results. As communication behaviors and patient perceptions can vary depending on the healthcare systems and cultural settings, the observed gender-related effects may not be directly generalizable to other countries. However, direct influence by the researchers on patient responses is unlikely, as the anesthetists who conducted the consultations were randomly assigned according to the schedule, had no knowledge of the allocation of patient groups, and followed a standardized procedure for preoperative documentation.

Second, the results are based on patient-reported measures, which are inherently subjective and may be influenced by individual expectations or perceptions. Finally, unmeasured factors—such as personal empathy, communication style, or the specific dynamics of each consultation—may have contributed to the observed differences and were not fully captured in this analysis.

Third, the study relied on patient-reported outcomes, such as the APAIS (Preoperative Anxiety and Information Scale) and Likert-scale ratings. Although these are validated instruments, they remain subjective and may be influenced by individual expectations, rapport with the anesthesiologist, or unmeasured psychosocial factors. These potential biases should be considered when interpreting the results. Despite these limitations, the strengths of the study include the relatively large cohort and the systematic assessment of both patient and anesthetist characteristics, which together provide valuable insights into preoperative preparation.

## Figures and Tables

**Table 1:** Descriptive table stratified by patient gender.

Number of Patients	Overall	Men	Women
	N = 275	183 (66.5%)	92 (33.5%)
Age-yr Mean (±SD)	54.6 (±15.4)	57.9 (±14.6)	48.0 (±14.8)
Median [Q1, Q3]	57.0 [43.0, 67.0]	60.0 [48.0, 68.0]	48.0 [35.0, 58.0]
Surgery risk			
Cardiosurgical	7 (3%)	6 (86%)	1 (14%)
High	6 (2%)	5 (83%)	1 (17%)
Intermediate	117 (44%)	87 (74%)	30 (26%)
Low	134 (51%)	81 (60%)	53 (40%)
Missing	11 (4%)	4 (36%)	7 (64%)
ASA * score			
Median [Q1, Q3]	2.0 [2.00, 2.00]	2.0 [2.00, 2.00]	2.0 [2.00, 2.00]
1	41 (15%)	22 (54%)	19 (46%)
2	178 (65%)	122 (69%)	56 (31%)
3	52 (19%)	36 (69%)	17 (33%)
4	2 (1%)	2 (100%)	0 (0%)
Missing	2 (1%)	1 (0.5%)	1 (1%)

* American Society of Anesthesiologists.

**Table 2:** Descriptive table stratified by anesthesiologist gender.

Number of Anesthetists	Overall	Men	Women
	N = 94	33 (35%)	61 (65%)
Clinical experience			
up to one year	28 (30%)	8 (29%)	20 (71%)
1 to 4 years	38 (40%)	13 (34%)	25 (66%)
more than 5 years	17 (18%)	6 (35%)	11 (65%)
more than 10 years	11 (12%)	6 (55%)	5 (45%)
Number of performed consultation visits	N = 275	94 (34%)	181 (66%)
up to one year	80 (29%)	22 (27.5%)	58 (72.5%)
1 to 4 years	94 (34%)	26 (28%)	68 (72%)
more than 5 years	52 (19%)	16 (31%)	36 (69%)
more than 10 years	49 (18%)	30 (61%)	19 (39%)

**Table 3:** Linear mixed model. Number of anesthesia-related risks explained to the patient during the preoperative consultation, analyzed by anesthesiologist gender and experience.

Number of Explained Anesthesia-Related Risks			
**Predictors**	**Estimates**	**Cl**	*p*
(Intercept)	6.12	3.12–9.13	<0.001
age	0.00	−0.04–0.04	0.979
sex patient [women]	−0.26	−1.58–1.07	0.704
sex premedication doctor [women]	0.03	−1.72–1.77	0.976
experience [1–4 years]	2.30	0.31–4.29	0.024
experience [>5 years]	1.49	−0.84–3.82	0.208
experience [>10 years]	2.36	−0.50–5.21	0.105
group [experimental]	0.11	−1.07–1.29	0.853
Random Effects			
σ2 16.10	ICC 0.30		
τ00 ANE 6.74	N ANE 88		
Observations	219		

**Table 4:** Linear mixed model. Number of risk recalls at two time points. The follow-up survey attains a value of 0 for measurement after consultation and a value of 1 for the online follow-up two days later.

Number of Risk Recall				
Predictors		Estimates	Cl	*p*
(Intercept)		12.40	10.24–14.57	<0.001
age		−0.04	−0.07–−0.01	0.005
sex patient [women]		−0.06	−1.05–0.94	0.913
sex premedication doctor [women]		−0.48	−1.47–0.50	0.336
experience [1–4 years]		0.64	−0.49–1.78	0.264
experience [>5 years]		1.22	−0.10–2.55	0.071
experience [>10 years]		0.07	−1.46–1.32	0.918
time		0.60	−0.20–1.39	0.141
group [experimental]		0.87	−0.19–1.94	0.107
time × group [experimental]		1.24	0.09–2.38	0.034
Random Effects				
σ2 9.19	τ00 ANE	0.00		
τ00 pat 8.48	N ANE	94		
N pat 275				
Observations		473		

**Table 5:** Linear mixed model. Fear level was measured on a four-point Likert scale on the consultation day.

Fear Level			
Predictors	Estimates	Cl	*p*
(Intercept)	3.27	2.90–3.63	<0.001
age	0.00	−0.00–0.01	0.078
sex patient [women]	0.00	−0.17–0.18	0.959
sex premedication doctor [women]	0.21	0.03–0.38	0.022
experience [1–4 years]	−0.01	−0.21–0.20	0.955
experience [>5 years]	−0.02	−0.25–0.22	0.896
experience [>10 years]	0.01	−0.26–0.27	0.962
group [experimental]	−0.01	−0.17–0.15	0.870
Random Effects			
σ2 0.37	ICC 0.02		
τ00 ANE 0.01	N ANE 91		
Observations	243		

**Table 6:** Linear mixed model. Communication quality is measured on a four-point Likert scale.

Communication Quality			
Predictors	Estimates	Cl	*p*
(Intercept)	0.53	0.42–0.64	<0.001
age	0.00	−0.00–0.00	0.609
sex patient [women]	−0.01	−0.07–0.04	0.596
sex premedication doctor [women]	−0.03	−0.09–0.02	0.240
experience [1–4 years]	0.04	0.03–0.11	0.233
experience [>5 years]	−0.01	−0.08–0.07	0.873
experience [>10 years]	0.09	0.00–0.17	0.049
group [experimental]	0.03	−0.02–0.08	0.259
Random Effects			
σ2 0.03	ICC 0.05		
τ00 ANE 0.00	N ANE 88		
Observations	229		

## Data Availability

The datasets presented in this article are not readily available because the data are part of an ongoing sub-study analysis. Requests to access the datasets should be directed to the corresponding author.

## Supplementary Materials

The following supporting information can be downloaded at https://www.mdpi.com/article/10.3390/jcm14217643/s1. Table S1. List of 15 anesthesia-related risk items. Table S2. Descriptive table stratified by patient gender. Overview of recognized risks. Table S3. Descriptive table stratified by patient gender. Six-point Amsterdam Preoperative Anxiety and Information Scale (APAIS). Table S4. Linear mixed model. The APAIS score, preoperative anesthesia-related anxiety and the need for information, on s scale from 1 to 5. Table S5. Descriptive table stratified by patient gender. Alleviated patient’s fears and concerns, and communication quality between patient and anaesthesiologist. Rated on 4-point Likert scale.

## Abbreviations

The following abbreviations are used in this manuscript:

ICT	Interactive consultation tool
APAIS	Preoperative Anxiety and Information Scale
LMMs	Linear mixed-effects models
Q1, Q3	Quartile 1 and quartile 3 define the lower and upper limits of the interquartile range
ASA	American Society of Anesthesiologists
