# Supplementary material for: The Influence of Anesthesiologist Gender and Experience on Risk Understanding and Anxiety Changes After Online Preoperative Patient Education: A Sub-Analysis of the iPREDICT Randomized Controlled Trial
Source: J Clin Med. 2025 Oct 28;14(21):7643. doi: 10.3390/jcm14217643 (PMC12609525; doi:10.3390/jcm14217643)
Supplement: Supplementary file 1 [file jcm-14-07643-s001.zip › jcm-3912613-supplementary.pdf]

Table S1. List of 15 anesthesia related risks items.

	**Anaesthesia related risks explained to the patient during the preoperative consultation**
**1**	Allergic reaction to administered medication
**2**	Bruising or nerve damage due to an injection or infusion and positioning damage
**3**	Infections at puncture sites or catheters
**4**	Low blood pressure
**5**	Blood loss, transfusion requirements
**6**	Thrombosis, embolism
**7**	Nausea and vomiting
**8**	Pneumonia
**9**	State of confusion, delirium
**10**	Tooth damage
**11**	Sore throat and hoarseness
**12**	Cramping of the airways
**13**	Waking up during the operation
**14**	Life-threatening rise in body temperature
**15**	Heart and breathing problems, brain damage and death

Table S2. *Descriptive table stratified by patient gender. Overview of risk recall at two times points.*

	Overall (N=275)	men (N=183)	women (N=92)
**Number of risks recalled on consultation day**			
Mean (±SD)	10.6 (± 4.80)	10.6 (± 4.75)	10.7 (± 4.84)
Median [Q1, Q3]	12.0 [8.00, 15.0]	12.0 [8.00, 14.0]	12.0 [9.00, 15.0]
Missing	32 (11.6%)	23 (12.6%)	9 (9.8%)
**Number of risks recalled, 2 days after consultation day**			
Mean (±SD)	11.7 (± 3.70)	11.5 (± 3.76)	12.2 (± 3.57)
Median [Q1, Q3]	13.0 [10.0, 15.0]	13.0 [10.0, 14.0]	13.0 [11.0, 15.0]
Missing	45 (16.4%)	33 (18.0%)	12 (13.0%)

Table S3. Descriptive table stratified by patient gender. Six-point Amsterdam Preoperative Anxiety and Information Scale (APAIS).

	Overall (N=275)	men (N=183)	women (N=92)
**APAIS score on consultation day**			
Mean (±SD)	2.12 (± 0.750)	2.03 (± 0.652)	2.30 (± 0.887)
Median [Q1, Q3]	2.00 [1.67, 2.33]	2.00 [1.67, 2.33]	2.33 [1.67, 2.67]
Missing	34 (12.4%)	24 (13.1%)	10 (10.9%)
**APAIS 1. I am worried about the anaesthetic.**			
Mean (±SD)	1.90 (± 0.896)	1.78 (± 0.816)	2.15 (± 0.995)
Median [Q1, Q3]	2.00 [1.00, 2.00]	2.00 [1.00, 2.00]	2.00 [1.00, 3.00]
Missing	34 (12.4%)	24 (13.1%)	10 (10.9%)
**APAIS 2. The anaesthetic is on my mind continually.**			
Mean (±SD)	1.71 (± 0.939)	1.56 (± 0.808)	2.00 (± 1.10)
Median [Q1, Q3]	1.00 [1.00, 2.00]	1.00 [1.00, 2.00]	2.00 [1.00, 3.00]
Missing	34 (12.4%)	24 (13.1%)	10 (10.9%)
**APAIS 3. I would like to know as much as possible about the anaesthetic.**			
Mean (±SD)	2.75 (± 1.05)	2.75 (± 1.00)	2.74 (± 1.14)
Median [Q1, Q3]	3.00 [2.00, 4.00]	3.00 [2.00, 4.00]	3.00 [2.00, 4.00]
Missing	34 (12.4%)	24 (13.1%)	10 (10.9%)

Table S4. Linear mixed model. The APAIS score, *preoperative* ***anesthesia-related*** *anxiety and the need for information, on s scale from 1 to 5.*

**APAIS score at consultation day**			
Predictors	Estimates	*Cl*	*p*
(Intercept)	2.40	1.96 – 2.84	**<0.001**
age	-0.01	-0.01 – -0.00	**0.036**
sex patient [women]	0.20	0.01 – 0.41	0.061
sex premedication doctor [women]	-0.11	-0.33 – 0.12	0.350
experience [1 - 4 years]	0.14	-0.12 – 0.39	0.303
experience [>5 years]	0.02	-0.28 – 0.31	0.903
experience [>10 years]	-0.02	-0.37 – 0.33	0.912
group [experimental]	0.04	-0.15 – 0.23	0.689
**Random Effects**			
σ^2 0.51	ICC 0.07		
τ_{00} ANE 0.04	N ANE 91		
Observations	241		

Table S5. Descriptive table stratified by patient gender. Alleviated patient's fears and concerns, and communication quality between patient and anaesthesiologist. Rated on 4-point Likert scale: 1-I do not agree, 2- I do rather not agree, 3-I rather agree, 4-I agree.

	**Overall (N=275)**	**men (N=183)**	**women (N=92)**
**Fear taken away**			
Mean (±SD)	3.65 (± 0.615)	3.66 (± 0.582)	3.61 (± 0.678)
Median [Q1, Q3]	4.00 [3.00, 4.00]	4.00 [3.00, 4.00]	4.00 [3.00, 4.00]
Missing	32 (11.6%)	23 (12.6%)	9 (9.8%)
**Effective communication**			
Mean (±SD)	3.66 (± 0.599)	3.67 (± 0.573)	3.62 (± 0.647)
Median [Q1, Q3]	4.00 [3.00, 4.00]	4.00 [3.00, 4.00]	4.00 [3.00, 4.00]
Missing	46 (16.7%)	33 (18.0%)	13 (14.1%)
**Patient asked questions**			
Mean (±SD)	2.31 (± 0.703)	2.29 (± 0.691)	2.33 (± 0.729)
Median [Q1, Q3]	2.00 [2.00, 3.00]	2.00 [2.00, 3.00]	2.00 [2.00, 3.00]
Missing	46 (16.7%)	33 (18.0%)	13 (14.1%)
**Need for information**			
Mean (±SD)	1.99 (± 0.775)	2.02 (± 0.773)	1.92 (± 0.781)
Median [Q1, Q3]	2.00 [1.00, 2.00]	2.00 [2.00, 2.00]	2.00 [1.00, 2.00]
Missing	46 (16.7%)	33 (18.0%)	13 (14.1%)
**Education refused**			
yes	89 (39 %)	59 (39 %)	30 (38 %)
no	140 (61 %)	91 (61 %)	49 (62 %)
Missing	46 (16.7%)	33 (18.0%)	13 (14.1%)
